# Phospholipid profiling identifies acyl chain elongation as a ubiquitous trait and potential target for the treatment of lung squamous cell carcinoma

**DOI:** 10.18632/oncotarget.7179

**Published:** 2016-02-03

**Authors:** Eyra Marien, Michael Meister, Thomas Muley, Teresa Gomez del Pulgar, Rita Derua, Jeffrey M. Spraggins, Raf Van de Plas, Frank Vanderhoydonc, Jelle Machiels, Maria Mercedes Binda, Jonas Dehairs, Jami Willette-Brown, Yinling Hu, Hendrik Dienemann, Michael Thomas, Philipp A. Schnabel, Richard M. Caprioli, Juan Carlos Lacal, Etienne Waelkens, Johannes V. Swinnen

**Affiliations:** ^1^ KU Leuven – University of Leuven, LKI - Leuven Cancer Institute, Department of Oncology, Laboratory of Lipid Metabolism and Cancer, Leuven, Belgium; ^2^ Thoraxklinik at University Hospital Heidelberg, Translational Research Unit, Heidelberg, Germany; ^3^ TLRC-H – Translational Lung Research Center Heidelberg, Member of The German Center for Lung Research, Heidelberg, Germany; ^4^ Fundación Jiménez Díaz, Division of Translational Oncology, Madrid, Spain; ^5^ KU Leuven – University of Leuven, Department of Cellular and Molecular Medicine, Laboratory of Protein Phosphorylation and Proteomics, Leuven, Belgium; ^6^ Vanderbilt University Medical Center, Department of Biochemistry and Mass Spectrometry Research Center, Nashville, TN, USA; ^7^ Delft University of Technology, Delft Center for Systems and Control, Delft, The Netherlands; ^8^ KU Leuven – University of Leuven, LKI - Leuven Cancer Institute, Department of Oncology, Abdominal Surgical Oncology, Leuven, Belgium; ^9^ National Cancer Institute, Centre for Cancer Research, Cancer and Inflammation Program, Frederick, MD, USA; ^10^ Thoraxklinik at University Hospital Heidelberg, Department of Surgery, Heidelberg, Germany; ^11^ Thoraxklinik at University Hospital Heidelberg, Department of Thoracic Oncology, Heidelberg, Germany; ^12^ University of The Saarland, Institut für Allgemeine und Spezielle Pathologie, Homburg/Saar, Germany

**Keywords:** lipidomics, phospholipids, cancer, ELOVL, lung SCC

## Abstract

Lung cancer is the leading cause of cancer death. Beyond first line treatment, few therapeutic options are available, particularly for squamous cell carcinoma (SCC). Here, we have explored the phospholipidomes of 30 human SCCs and found that they almost invariably (in 96.7% of cases) contain phospholipids with longer acyl chains compared to matched normal tissues. This trait was confirmed using *in situ* 2D-imaging MS on tissue sections and by phospholipidomics of tumor and normal lung tissue of the *L-Ikkα^KA/KA^* mouse model of lung SCC. In both human and mouse, the increase in acyl chain length in cancer tissue was accompanied by significant changes in the expression of acyl chain elongases (ELOVLs). Functional screening of differentially expressed ELOVLs by selective gene knockdown in SCC cell lines followed by phospholipidomics revealed ELOVL6 as the main elongation enzyme responsible for acyl chain elongation in cancer cells. Interestingly, inhibition of ELOVL6 drastically reduced colony formation of multiple SCC cell lines *in vitro* and significantly attenuated their growth as xenografts *in vivo* in mouse models. These findings identify acyl chain elongation as one of the most common traits of lung SCC discovered so far and pinpoint ELOVL6 as a novel potential target for cancer intervention.

## INTRODUCTION

Lung cancer is the deadliest type of cancer for both men and women [1]. Beyond the first line of therapy, few effective therapeutic options are available, particularly for lung squamous cell carcinoma (SCC), which develops from the flat, surface-covering cells in the airways, and is one of the major lung cancer subtypes induced by smoking. So far, most efforts towards the identification of novel targets have focused on the molecular characterization of lung cancer at the level of the genome and transcriptome [2–5]. Several of these studies have emphasized the heterogeneity of lung cancers characterized by scattered genetic traits and transcriptomic subtypes. The lipidome and particularly the phospholipidome remain, however, largely unexplored. Nevertheless, the phospholipidome takes a unique position in the omics landscape. Situated at the far end of the gene regulatory cascade, the phospholipidome integrates biological information at several levels, including genetics, gene expression, protein activity and environmental/dietary cues. Being essential for the formation of membranes, the phospholipidome in turn affects numerous cellular processes and therefore is more closely related to the cellular pathophysiology than almost any other ‘ome’. Hence the phospholipidome may be a rich, yet, underexplored source of molecular and biochemical information to find cancer traits that are more ubiquitous and that may be exploited in larger cohorts of cancer patients. Recent analysis of the phospholipidome of lung cancer tissues has revealed marked alterations in multiple individual phospholipid species upon tumor development [6]. Here, to gain more insight into these changes and to identify specific pathways and enzymes driving these changes, we have further mined these data and have performed functional screenings by selective gene knockdown. These analyses reveal that acyl chain elongation is one of the most prominent pathways affected in lung SCC and pinpoint ELOVL6 as one of the main drivers of these changes and as potential target for antineoplastic intervention.

## RESULTS

### Mining of phospholipid profiles of lung SCC reveals the presence of longer phospholipids in clinical tumor samples

**Figure 1:**
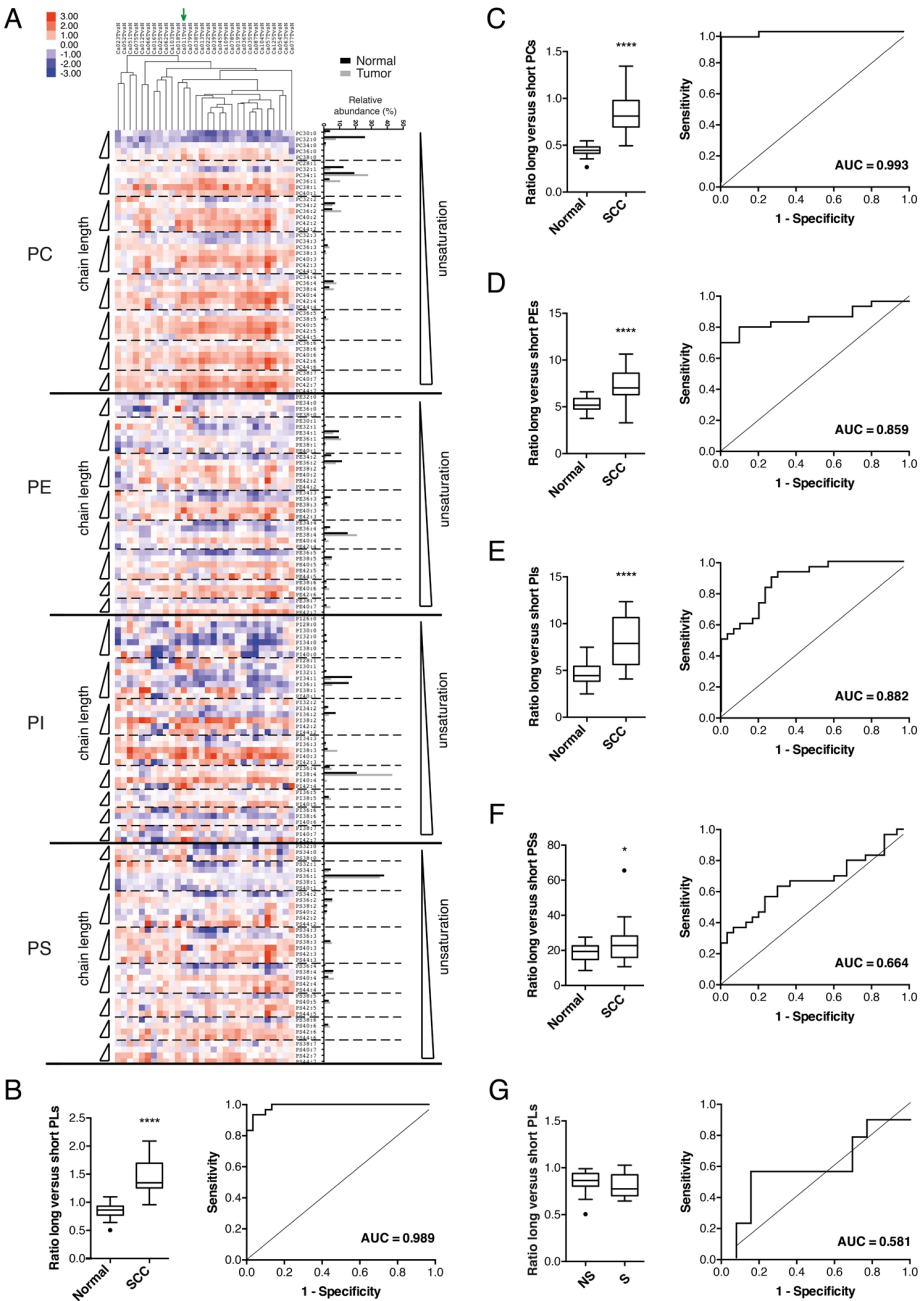
Changes in phospholipid profiles in clinical samples of human lung SCC tumor versus matched normal lung tissue **A**. The relative abundance of individual phospholipid species was measured in lipid extracts from tumor and matched normal tissue from 30 SCC patients using ESI-MS/MS operated in MRM mode. Phospholipids are ordered according to headgroup class (phosphatidylcholine (PC), phosphatidylethanolamine (PE), phosphatidylinositol (PI) and phosphatidylserine (PS)). Within each headgroup class, species are ranked according to the degree of unsaturation (separated from each other by a dotted line) and within each subclass of (un)saturation according to their chain length. Blue squares indicate a decrease in relative phospholipid abundance while red squares represent an increase as indicated by the scale bar (log2 of the ratio). Grey squares indicate missing values. The green arrow indicates the primary tumor from which the 2427PT cell line was derived. On the right the relative abundance of each phospholipid species is shown for a representative SCC tumor and matched normal tissue. Graphs (B-F) show the combined acyl chain length index and corresponding ROC curve and AUC for 30 lung SCCs versus matched normal tissues. The acyl chain length index is calculated as the ratio of the total abundance of **B**. all phospholipid species (PLs), **C**. PCs, **D**. PEs, **E**. PIs, and **F**. PSs with longer fatty acyl chains (≥ 36 carbon atoms in the two acyl chains together) versus species with shorter fatty acyl chains (≤ 34 carbon atoms). * *p* < 0.05; **** *p* < 0.0001 (Student's test/Wilcoxon test). In graph **G**., the acyl chain length index (based on all PLs) is calculated for normal tissues from patients who have quit smoking for more than 6 months (NS; n=13) and smokers (S; n=9) at the time of surgery. The corresponding ROC curve and AUC are given. (Mann-Whitney test).

Phospholipid profiles of tumor and matched normal tissue from 30 lung SCC patients (see Supplementary Table S1 for patient characteristics) [6] were subjected to a more extensive data analysis and heat mapping to reveal any patterns of changes and to uncover alterations in specific lipid-related pathways. Supervised ranking of lipid species according to their combined acyl chain length revealed a dramatic and recurrent increase in the relative abundance of phospholipid species with longer acyl chains (larger total number of acyl chain carbon atoms) at the expense of shorter species in the four phospholipid headgroup class that were analyzed (phosphatidylcholine (PC), phosphatidylethanolamine (PE), phosphatidylinositol (PI) and phosphatidylserine (PS)) (Figure 1A). To better quantify these changes we have calculated a combined acyl chain length index by comparing the total abundance of phospholipid species with longer fatty acyl chains (≥ 36 carbon atoms in the two acyl chains together) versus species with shorter fatty acyl chains (≤ 34 carbon atoms). When applied for all phospholipid species together, a significantly higher value was found in SCC tumor tissue compared to normal lung tissue (AUC = 0.989; *p*<0.0001; Figure 1B). For an acyl chain length index set at 1, longer acyl chains were found in 96.7% of SCC, with a corresponding specificity of 90.0% (Figure 1B). Calculation of the acyl chain length index for each phospholipid headgroup class separately, revealed a similar trend (Figure 1C-1F), with PC being the most affected with an almost perfect correlation (AUC = 0.993; *p*<0.0001) of acyl chain length index and tumor tissue identification (Figure 1C). To exclude that the observed differences in acyl chain length simply reflect smoking behavior, we have compared the acyl chain length index of normal tissues from smokers versus patients who have quit smoking for more than 6 months. No difference was observed (Figure 1G).

**Figure 2:**
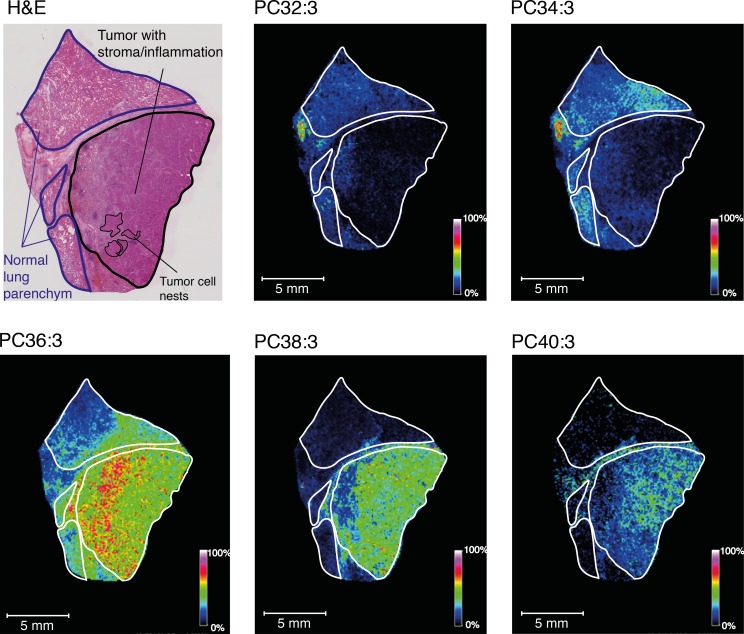
2D-Imaging MS of a series of phospholipids differing in combined acyl chain length in a representative SCC and adjacent non-malignant tissue section MALDI FTICR MS ion images of the selected molecular ions PC32:3 (*m/z* 728.5214 Da ± 0.004, 1.5 ppm), PC34:3 (*m/z* 756.5537 Da ± 0.004, 0.13 ppm), PC36:3 (*m/z* 784.5854 Da ± 0.004, 0.38 ppm), PC38:3 (*m/z* 812.6162 Da ± 0.004, 0.25 ppm) and PC40:3 (*m/z* 840.6485 Da ± 0.004, 0.95 ppm) in a representative SCC and adjacent non-malignant tissue. Color intensities vary between 0 and 100% as indicated by the scale bars. H&E staining of a next tissue section is shown. Normal tissue, tumor tissue with stroma/inflammation, and tumor cell nests are indicated. Note that not all tumor cell nests are indicated, but some are circled as examples.

To corroborate these findings and to unequivocally demonstrate that phospholipids with longer acyl chains are more prominent in cancer tissue compared to normal tissue, we performed 2D-imaging mass spectrometry (MS) of a representative series of phospholipids differing in combined acyl chain length (PC32:3, PC34:3, PC36:3, PC38:3 and PC40:3) in tissue sections containing both cancer and adjacent non-malignant tissue. This analysis revealed that the shorter species (PC32:3 and PC34:3) were predominant in normal tissue, whereas the longer species (PC36:3, PC38:3 and PC40:3) were mainly found in cancer regions of the tissue sections (Figure 2). High signals of PC36:3 and PC38:3 were found in both tumor nests and associated stroma. The longest phospholipid (PC40:3) was more defined to the tumor nests. These findings revealed that tumor tissue and particularly tumor cells have longer phospholipids than the non-malignant counterparts.

### Phospholipids with long acyl chains are also found in the IKKα knock-in mouse model of lung SCC

**Figure 3:**
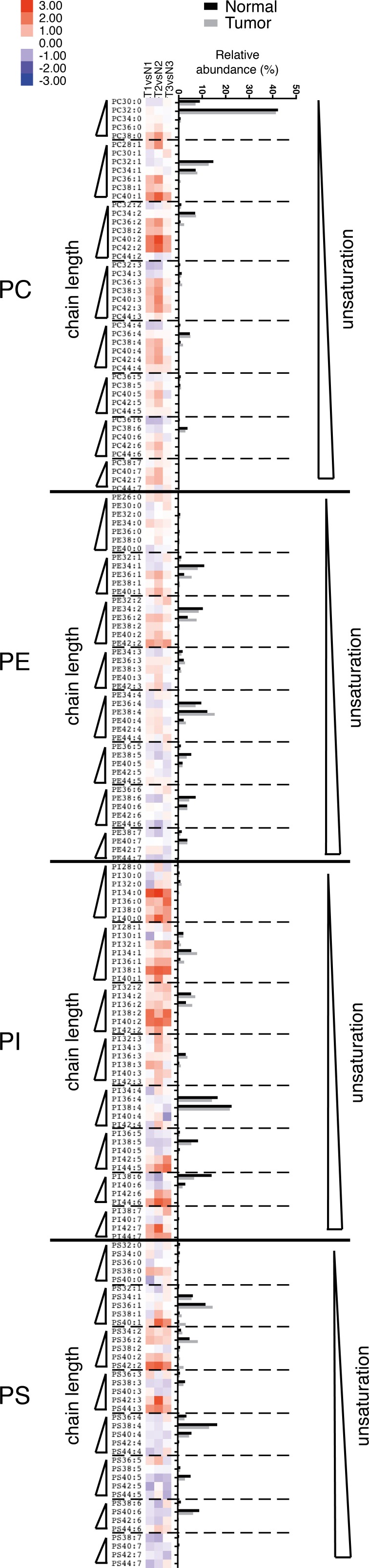
Presence of phospholipids with longer acyl chains in the L-IkkαKA/KA SCC mouse model The relative abundance of phospholipids was measured in extracts from tumor and matched normal tissue from *L-Ikkα^KA/KA^* SCC mice (n=3) using ESI-MS/MS operated in MRM mode. The heatmap is organized as explained in the legend to Figure 1.

To demonstrate that changes in acyl chain length of phospholipids are more universally linked to lung SCC development, we sought to confirm these findings in a lung SCC mouse model. The model that we explored is based on a knock-in of kinase-dead IKKα (IκB Kinase α) and spontaneously develops SCC, closely resembling human SCC [7]. Intriguingly, very similar changes in phospholipid composition were observed as in human clinical tissues, with tumor tissues showing longer phospholipids than healthy tissues (Figure 3).

### The increase in longer phospholipids in tumor tissue is caused by acyl chain elongation and is mediated by ELOVL6

**Figure 4:**
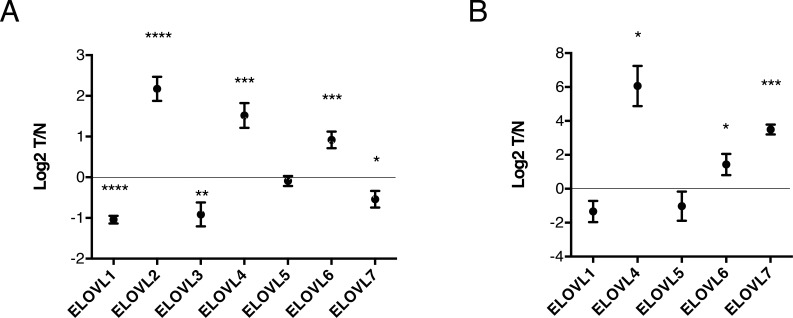
Expression profiling of ELOVLs in human and mouse lung SCC **A**. mRNA levels of ELOVL1-7 were analyzed by RT-qPCR in 30 human SCC tissues and 9 pools (14-15 different samples) of matched normal tissues. A normalization factor corresponding to the geometric mean of Ct values of ESD, HMBS, POLR2A, PSMB4, TBP and UBC was used for subsequent ΔCt calculation. **B**. Tumor versus normal lung tissue (n=3) from the *L-IKKα^KA/KA^* SCC mouse model were collected and mRNA levels of ELOVL1-7 were measured using RT-qPCR. 18S was used for normalization. ELOVL2 and 3 were not detected. Data represent mean (log2) ± standard error. * *p* < 0.05; ** *p* < 0.01; *** *p* < 0.001; **** *p* < 0.0001 (Student's test).

**Figure 5:**
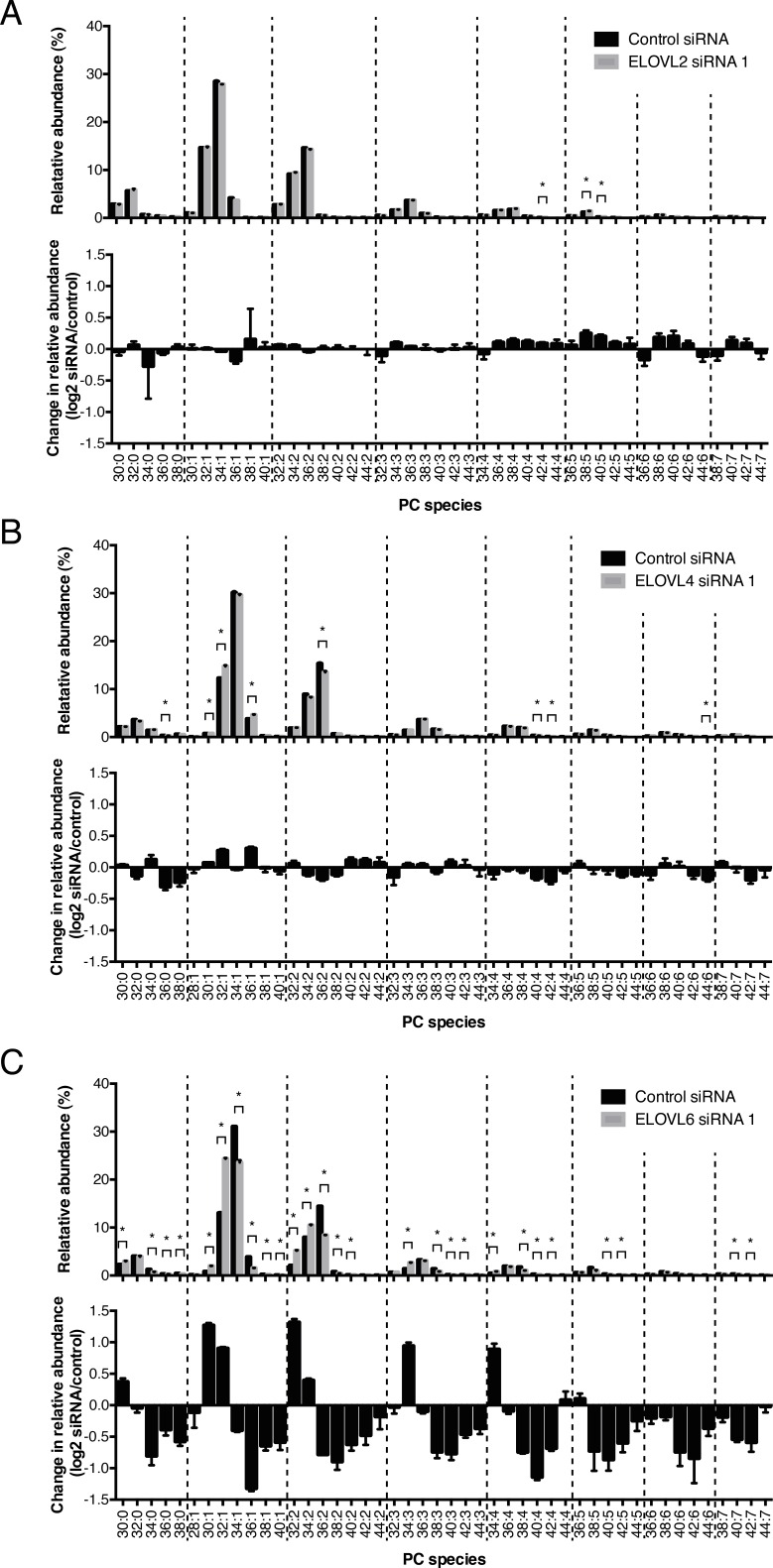
Effect of knockdown of ELOVL2, 4 and 6 on acyl chain elongation in 2427PT lung SCC cells 2427PT cells were transfected with siRNA specific for **A**. ELOVL2, **B**. ELOVL4 and **C**. ELOVL6 for 72h. Control cells were transfected with scrambled siRNA. The relative abundance and changes in relative abundance (log2) of PC after modulation of the specific ELOVL are shown. Lipid extracts were made and were subjected to MRM-based ESI-MS/MS analysis of phospholipids. Lipid species are grouped based on degree of fatty acyl chain unsaturation (groups separated by dotted lines). In each group with equal (un)saturations, the species are ordered from species with shorter fatty acyl chains to longer chains. Data represent mean (n=3) ± standard deviation. * Indicates statistical significance after multiple testing using the Holm-Sidak method with alpha=0.05.

Next, we explored the mechanism underlying the increase in longer chain phospholipids in cancer tissues. Acyl chain length of lipids is largely regulated by the process of fatty acid elongation, which is catalyzed by a complex enzyme system of which ELOVLs (elongation of very long fatty acids) commit the first and rate-limiting step by adding 2-carbon units from malonyl-CoA to a given fatty acid. To date, seven distinct ELOVLs have been identified (ELOVL1-ELOVL7) [8]. To explore potential changes in the expression of these enzymes in lung SCC, we performed reverse transcription quantitative PCR (RT-qPCR) analysis of all 7 ELOVLs in tumor and pooled normal tissues of the same patient cohort. Significant changes in the expression of several ELOVLs were observed, with ELOVL1, 3 and 7 going down in SCC and ELOVLs 2, 4 and 6 going up (Figure 4A). Intriguingly, a very similar change in ELOVL expression profile was observed in the lung SCC mouse model (Figure 4B). Next, to explore to which extent these different ELOVLs contribute to the observed increase in acyl chain elongation in SCC, we knocked down the various ELOVLs that were increased in SCC in our qPCR analysis, using two independent siRNAs and assessed the effect on acyl chain elongation by MS-based lipidomics. To this end we used the previously established 2427PT cell line [9], which was derived from patient Ca011, whose primary tumor was included in our lipidomics analysis in Figure 1A (indicated by a green arrow) and who showed a pronounced elongation phenotype. As shown in Supplementary Figure S1, knockdown efficiency was at least 85% and all siRNAs were selective for a specific ELOVL. Interestingly, only ELOVL6 knockdown completely reversed the elongation profile observed in SCC (shown for PC in Figure 5 (siRNA 1) and Supplementary Figure S2 (siRNA 2)).

**Figure 6:**
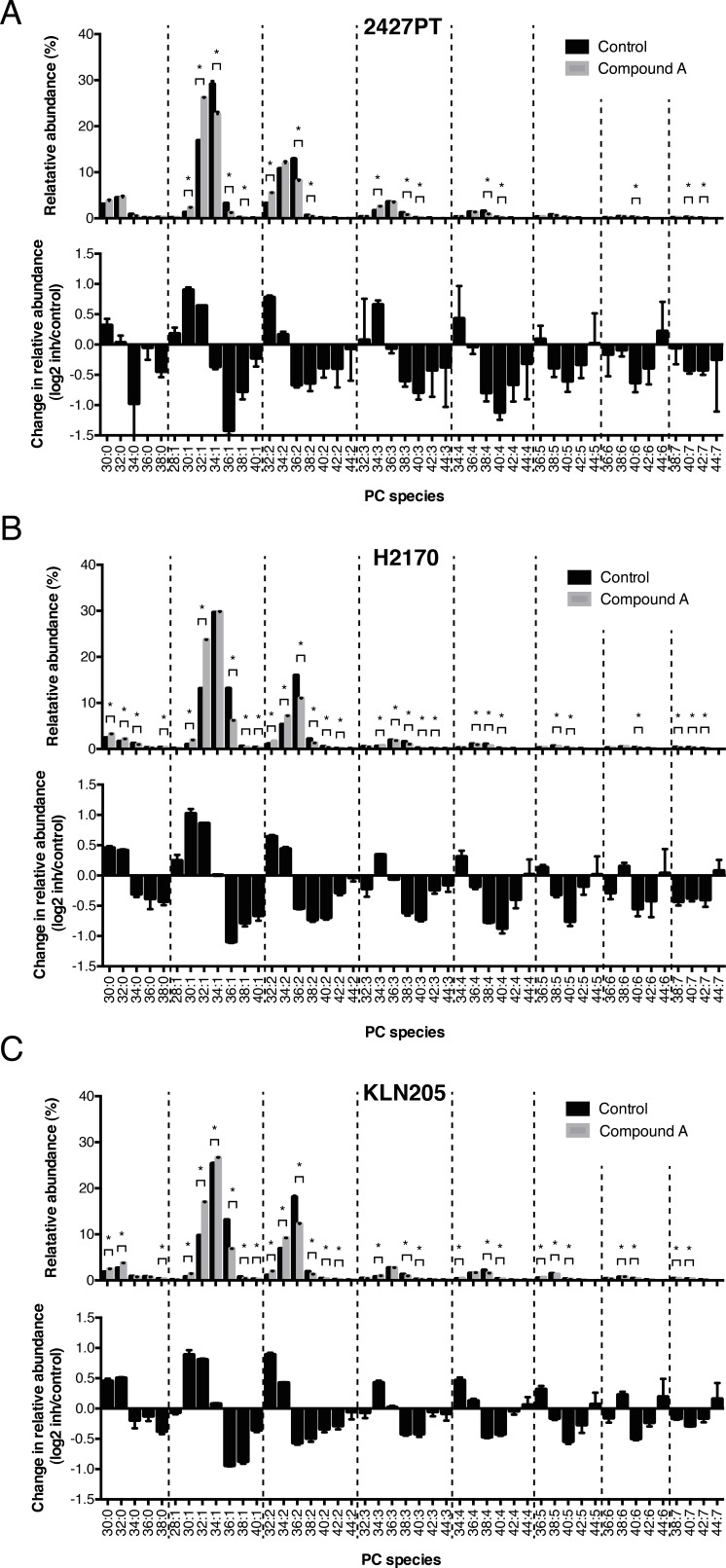
Reversal of the elongation phenotype by chemical inhibition of ELOVL6 in lung SCC cells **A**. 2427PT, **B**. H2170 and **C**. KLN205 cells were treated with the ELOVL6 inhibitor Compound A (1 μM) for 72h and control cells were treated with vehicle. The relative abundance and changes in relative abundance (log2) of PC after ELOVL6 inhibition are shown. Changes in lipids were analyzed and presented as in Figure 5. Data represent mean (n=3) ± standard deviation. * Indicates statistical significance after multiple testing using the Holm-Sidak method with alpha=0.05.

**Figure 7:**
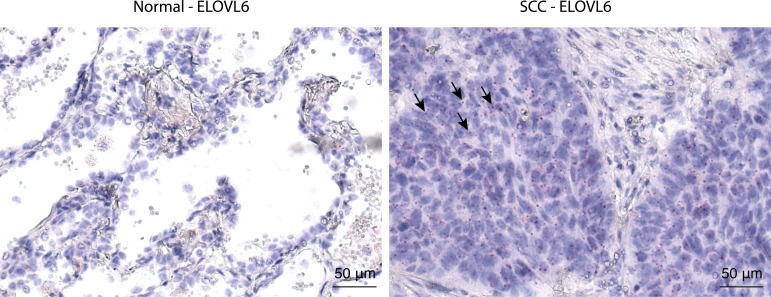
ISH staining for ELOVL6 ISH staining for ELOVL6 using the RNAscope technology on a tumor and matched normal tissue section from a representative SCC patient. Every red dot represents one mRNA molecule of ELOVL6. Some red dots are indicated by arrows as examples.

Silencing of ELOVL6 using siRNA showed identical effects in another established human SCC cell line H2170 (Supplementary Figure S3). Similar changes were observed when these and also a mouse SCC cell line, KLN205, were treated with a selective chemical ELOVL6 inhibitor, Compound A [10] (shown for PC in Figure 6). As ELOVL6 has a strong substrate specificity for C16:0 and C16:1, which it converts to C18:0 and C18:1 [8], respectively, we analyzed the differences in acyl chain composition of phospholipids in cancer versus non-malignant tissue in more detail by tandem MS. This analysis confirmed that tumor tissue contained phospholipid species with a higher content of C18:0 and C18:1 at the expense of C16:0 and C16:1 compared to normal tissue (Supplementary Figure S4). To confirm that ELOVL6 expression is mainly confined to the cancer cells in clinical SCC tissue, we used RNAscope as no specific antibodies against ELOVL6 are available and also the generation of own antibodies failed. ELOVL6 expression was indeed found mainly in tumor cells, whereas in normal lung cells, almost no ELOVL6 expression was observed (Figure 7). Collectively, these results strongly indicate that ELOVL6 plays a key role in the overall elongation phenotype found in lung SCC.

### ELOVL6 inhibition attenuates tumor growth

**Figure 8:**
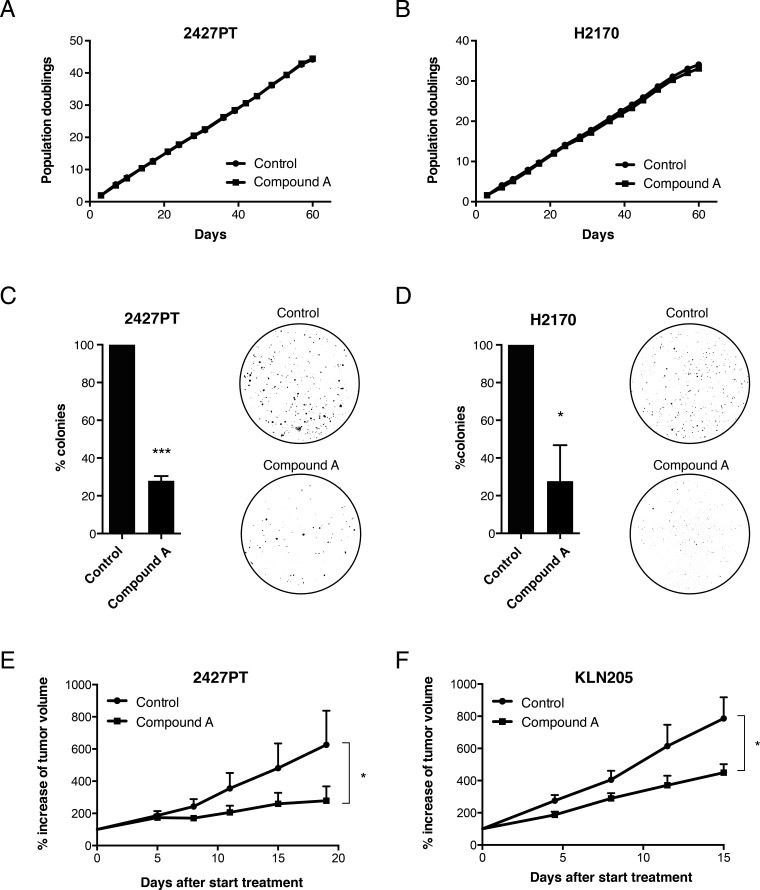
Effect of ELOVL6 inhibition on tumor growth The SCC cell lines **A**. 2427PT and **B**. H2170 were cultured as monolayers and were treated with Compound A (1 μM) or vehicle for 60 days. Graphs show the cumulative number of population doublings. The SCC cell lines **C**. 2427PT and **D**. H2170 were grown in soft agar for 21 days and were treated with Compound A (1 μM) or vehicle. Graphs show the percentage of anchor-independent growth for 2427PT and H2170 cells when treated with ELOVL6 inhibitor versus control cells. Data represent mean (n=3) ± standard deviation. * *p* < 0.05; *** *p* < 0.001 (Student's test). **E**. The SCC cell line 2427PT was subcutaneously injected in NMRI nu/nu mice. After 3 weeks, twice daily administration of Compound A was started versus vehicle. Tumor growth was monitored via caliper measurement twice a week. Data represent mean (n=9-10) ± standard error. * *p* < 0.05 (Two-way ANOVA with repeated measurements – Sidak's multiple comparisons test). **F**. The SCC cell line KLN205 was subcutaneously injected in syngeneic DBA/2 mice. Once tumor volume reached ±150 mm^3^ daily intra-tumoral injection of Compound A was started versus vehicle treated mice. Data represent mean (n=6) ± standard error. * *p* < 0.05 (Two-way ANOVA with repeated measurements – Sidak's multiple comparisons test).

To investigate whether chemical inhibition of ELOVL6 may have potential for therapeutic intervention, we examined the impact of Compound A on tumor growth *in vitro* and in mouse models *in vivo.* Surprisingly, despite the major changes in lipid composition, inhibition of ELOVL6 by Compound A did not result in any significant difference in cell proliferation, both in 2427PT cells and in H2170 cells grown as a monolayer on a solid surface (Figure 8A and 8B). However, in colony formation tests in soft agar, Compound A-treated cells showed a dramatic decrease in colony formation compared to vehicle-treated cells for both cell lines (Figure 8C and 8D). In addition, daily systemic administration of Compound A by oral gavage significantly attenuated tumor growth in nude mice bearing 2427PT-derived subcutaneous xenografts (Figure 8E). Moreover, also local chemical inhibition of ELOVL6, via intra-tumoral injection of Compound A, significantly attenuated tumor formation by KLN205 SCC cells grown as subcutaneous xenografts in syngeneic DBA/2 mice (Figure 8F).

## DISCUSSION

Despite important advances in treatments, lung cancer remains one of the most deadly cancers. To get a better handle on this disease, novel targeted approaches are highly needed, particularly for lung SCC, for which few approved targeted therapies are available. Here, we have mined the phospholipidome of SCC tissue and matched normal tissue in a search for novel potentially exploitable key traits of lung SCC. Interestingly, this analysis revealed acyl chain elongation of phospholipids as a very common characteristic of lung SCC tumors. In fact, acyl chain elongation was found in 96.7% of SCCs, making it perhaps the most common cancer trait discovered so far. Interestingly, similar changes were observed in spontaneously developing lung SCC tumors in the IKKα mouse model, suggesting that acyl chain elongation is a ubiquitous and fundamental event accompanying lung SCC development and pleating for faithfulness in this lung SCC model. Interestingly, increased acyl chain elongation has also been reported in nonalcoholic steatohepatitis (NASH)-associated hepatocellular carcinoma [11, 12], but as far as we are aware, this is the first study showing such an extensive alteration in acyl chain elongation in lung tumors.

Changes in acyl chain length were accompanied by significant changes in the expression of several ELOVLs with ELOVLs 1, 3 and 7 being down-regulated and ELOVLs 2, 4 and 6 being up-regulated in cancer samples. A very similar change in ELOVL expression profile was observed in an independent patient cohort present in the Oncomine database (Supplementary Figure S5A). To further explore whether ELOVLs are linked with any clinicopathological features, we analyzed the correlation of ELOVL expression with clinical stage. Intriguingly, a significant association was found between ELOVL4 and ELOVL6 expression with clinical stage (Supplementary Figure S5B). Also in the literature, evidence is accumulating that ELOVLs are often affected in cancer cells. ELOVL1 is highly expressed in clinical breast cancer samples [13]. ELOVL2 was found to be up-regulated in hepatocellular carcinoma [14]. ELOVL4 seems to be down-regulated in pancreatic cancer [15] and in hepatocellular carcinoma [16]. Also ELOVL5 is frequently down-regulated [17]. ELOVL6 is overexpressed in NASH-associated hepatocellular carcinoma [11, 12, 18] and in acute myeloid leukemia, while ELOVL7 is overexpressed in prostate cancer cells. The mechanisms of these changes may be very diverse. *ELOVL4* has been shown to be methylated in pancreatic cancer [15], whereas there is loss of copy number in hepatocellular carcinoma [16]. In acute myeloid leukemia ELOVL6 appears to be activated by a novel chromosome rearrangement causing the activation of both *EGF* and *ELOVL6* [19]. The mechanisms underlying the observed changes in ELOVLs in SCC in the current study remain to be explored. In the mouse model we used, changes in the expression of ELOVLs appears to be indirectly driven by loss of IKKα as it's kinase death form is expressed in both malignant and benign lung tissue, suggesting that other accompanying alterations in tumor suppressors and oncogenes drive these changes. These may include MYC [20, 21] and TP53 [22]. Interestingly, IKKα, which is also often down-regulated in human lung SCCs [23, 24] is known to suppress MYC-related pathways in adult mice [25–28]. *MYC* is often amplified in lung SCC [20, 21] and we found evidence from the UCSC genome browser that ELOVL6 expression is controlled by MYC (Supplementary Figure S6). TP53, which is often mutated in lung cancer [22], was recently shown to repress the transcription factor SREBP1c and accordingly several ELOVLs [29]. Besides changes in the expression of ELOVLs, an increase in substrate availability could drive the elongation process in tumors. In this context, the acetate dependence of tumors is recently gaining interest [30, 31]. Acetyl-CoA synthetase 2, which is often up-regulated in tumors, including lung tumors [30], ligates acetate and CoA producing acetyl-CoA. Acetyl-CoA can further be metabolized via acetyl-CoA carboxylase (ACACA) to malonyl-CoA, which is the substrate for fatty acid elongation. It has been shown that inhibition of ACACA using the chemical inhibitor soraphen interferes with elongation [32], which corroborates this hypothesis. Also LKB1, which is decreased in human lung cancer [33] as well as in the *L-Ikkα^KA/KA^* SCC mouse model [7], may shuttle substrates into the elongation pathway by regulating ACACA. In addition, increased substrate concentration can also be caused by an increased glycolytic flux in cancer cells, including lung cancers [34]. Intermediates that branch from glycolysis can be fueled into lipid precursors. These observations support our concept on the phospholipidome as an integrator of many signals.

Using functional screenings by selective gene knockdown of the three ELOVLs that are overexpressed in clinical lung SCC (ELOVLs 2, 4 and 6), we have provided evidence that ELOVL6 is the main enzyme responsible for the generation of longer lipids in lung SCC, despite the finding that is not the most dramatically overexpressed. Knockdown or inhibition of ELOVL6 in several lung SCC cell line models completely reversed the elongation phenotype. Moreover, in line with the substrate specificity of ELOVL6 for C16:0 and C16:1, we discovered that tumor tissue contained phospholipid species with a higher content of C18:0 and C18:1 at the expense of C16:0 and C16:1 compared to normal tissue.

Importantly, our findings indicate that reversal of the elongation phenotype by inhibition of ELOVL6 affects cancer biology and may have potential for antineoplastic intervention. In earlier reports it has been shown that silencing of ELOVL1 reduced cell viability of breast cancer cells significantly [13] and that inhibition of ELOVL7 resulted in drastic attenuation of prostate cancer cell growth [35]. Here we show that inhibition of ELOVL6 affects colony formation in soft agar, and tumor growth *in vivo* but not in monolayer, suggesting that mechanisms of anchorage-independent growth are affected by ELOVL6-mediated acyl chain elongation. Although the underlying mechanism remains to be explored, it is tempting to speculate that acyl chain elongation affects local membrane structure and thickness and may influence the topology and/or activity of membrane proteins by modulating hydrophobic mismatch, folding or structure [36–38]. *In vivo*, additional effects may be involved. Interesting in this context are the recent observations that ELOVL6 promotes inflammation by C18-mediated lipotoxicity and immune interaction in NASH [11, 18, 39], diet-induced insulin resistance [40–42] and atherosclerosis [43].

In the current study we show that chemical inhibition of ELOVL6 by Compound A interferes with tumor growth *in vivo* in two independent models. As Compound A is not highly optimized for use *in vivo*, this study suggests that ELOVL6 has significant potential as target for cancer intervention and sparks the interest in finding better chemical inhibitors with improved *in vivo* efficacy that could reverse the elongation phenotype in tumor tissue. Importantly, despite the dramatic alterations in fatty acid and corresponding phospholipid composition, *Elovl6^−/−^* mice appear grossly normal and viable [40–42], demonstrating that ELOVL6 is not essential for the development and functioning of normal tissues, suggesting that there is a broad therapeutic window for ELOVL6 inhibition in the context of cancer treatment.

In summary, our findings indicate that acyl chain elongation is one of the most common traits of lung SCCs and may actively contribute to cancer development. These findings suggest that inhibition of acyl chain elongation may represent a novel therapeutic approach to target a broad range of lung SCC patients. Moreover, lipid profiling and particularly analysis of acyl chain elongation profiles may find applications in future diagnostic platforms.

## MATERIALS AND METHODS

### Clinical tissue specimens

Tumor and matched distant (> 5 cm) normal lung tissue samples were collected from 30 SCC patients undergoing surgery as well as from an additional cohort of 31 SCCs (Supplementary Table S1). Selection was based on stage (I-IIIa), complete resection of the tumor during surgery, absence of prior malignancy, viable tumor content of ≥50%, and macroscopically confirmed absence of tumor in matched normal tissue as previously described [6]. Consecutive tissue sections were prepared using a cryotome for lipid analysis and qPCR. All patients gave their informed consent following the guidelines of the 2008 revision of the declaration of Helsinki and the local Ethics Committee of the Medical Faculty Heidelberg. Approval to perform lipidomics analysis on clinical samples was obtained from the local Ethical Committee of the KU Leuven.

### Mouse tissue specimens

Tumor and adjacent normal tissue from 68-week-old female Lori.IKKα;*Ikkα^KA/KA^* (*L-Ikkα^KA/KA^*) mice with FVB background were collected at the National Cancer Institute (Frederick, Maryland, USA) [7]. Mice were euthanized, tumor and matched normal lung tissues were dissected and washed in PBS prior to snap-freezing in liquid nitrogen and storage at −80°C.

### Cell culture and treatments

H2170 human and KLN205 mouse SCC cells were obtained from the American Type Culture Collection (ATCC; Manassas, Virginia, USA; December 2012) and from The European Collection of Cell Cultures (ECACC; Public Health England, Salisbury, UK; January 2014), respectively, and were cultured in RPMI1640 (Life Technologies, Carlsbad, California, USA). Cell experiments were performed fewer than 6 months after receipt of both cell lines. 2427PT human SCC cells were developed at the Thoraxklinik (UHEI; Heidelberg, Germany) [9] and cultured in DMEM/F-12 (Life Technologies). All media were supplemented with 10% fetal bovine Serum (FBS; Life Technologies) and 2 mM L-glutamine (Life Technologies) and cells were kept at 37°C in a humidified atmosphere of 5% CO_2_. For modulation of ELOVLs by RNA interference, cells were transfected with commercially available and validated siRNAs for ELOVL2 (siRNA 1: J_009531_10; siRNA 2: J_009531_11; GE Dharmacon, Lafayette, Colorado, USA), ELOVL4 (siRNA 1: s13558; siRNA 2: s13559; Life Technologies), ELOVL6 (siRNA1: s35516; siRNA2: s35518; Life Technologies) and corresponding control siRNA using Lipofectamine RNAiMAX (Life Technologies) as transfection reagent. For chemical inhibition of ELOVL6, Compound A [10] was obtained from the Centre for Drug Design and Discovery (CD3; Leuven, Belgium) and added to culture media from a 1000-fold stock solution in DMSO.

### Proliferation experiments

Cells were cultured in T25 flasks and treated with ELOVL6 inhibitor (1 μM) or vehicle (DMSO; 0.1% final concentration) for 60 consecutive days with intermittent passaging, medium replacement and cell counting twice a week. The number of population doublings was calculated using the formula (log n_t1_ – log n_t0_)/log2 with n_t0_ = number of cells when seeded and n_t1_ = number of cells counted at the moment of splitting.

### Anchorage-independent growth

For anchor-independent growth, cells were plated in a layer of 0.3% agar, which was put on top of a bottom layer of 0.5% agar. The soft agar phase was covered with 1 ml of medium with ELOVL6 inhibitor (1 μM) or vehicle (DMSO; 0.1% final concentration) and was replaced 3 times a week. After 21 days, plates were stained with 0.005% crystal violet. Colonies were counted using Image J software (National Institute of Health, Bethesda, Maryland, USA) [44].

### *In vivo* tumor growth

2427PT cells (3.5 10^5^ cells in 200 μl PBS/matrigel (1:1 v/v; Becton Dickinson, Heidelberg, Germany)) were subcutaneously injected in 7-week old female homozygous Naval Medical Research Institute nude mice (NMRI nu/nu; Charles River, Sulzfeld, Germany). 3 weeks after injection, oral administration of Compound A (30 mg/kg in 0.1% (v/v) Tween80 and 0.5% (w/v) carboxymethylcellulose in water (v/v)) via oral gavaging twice a day was started for 3 weeks. Control mice were treated twice daily with vehicle only.

KLN205 cells (5 10^5^ cells in 100 μl PBS) were subcutaneously injected in 9-week-old male DBA/2 mice (Janvier, Le Genest St. Isle, France). Once tumor volume was approximately 150 mm^3^ daily intra-tumoral injection of ELOVL6 inhibitor (10 μM in PBS) versus control (DMSO in PBS; 0.1% final concentration) was started for 14 days.

Tumors were measured twice a week using a caliper when mice were anesthetized (1.5-2% isoflurane in 100% oxygen). The tumor volume was estimated via the formula 0,52 x a x b^2^ (with a = largest diameter and b = smallest diameter).

### Phospholipid profiling

Lipids were extracted and phospholipid profiling was performed using a shotgun electrospray ionization tandem mass spectrometry (ESI-MS/MS) operated in multiple reaction monitoring (MRM) mode as described previously [6]. Clustering analysis was carried out using an average linkage-clustering algorithm (Spearman rank correlation) in the Cluster 3.0 software (Human Genome Centre, University of Tokyo, Tokyo, Japan) [45]. The clustering results were visualized using the Java TreeView 1.1.5 software (Stanford University School of Medicine, Stanford, California, USA) [46]. The molecular composition of selected PC species was characterized by MS^3^ fragmentation on a hybrid triple quadrupole/linear ion trap mass spectrometer (4000 QTRAP; AB SCIEX, Framingham, Massachusetts, USA) essentially as described in Ekroos *et al.* [47]. To this aim, lipid pellets were reconstituted in CHCl_3_:CH_3_OH 1:2 (v/v) containing 5 mM ammonium acetate.

### Reverse transcription quantitative PCR (RT-qPCR)

For patient material, RNA was isolated using the ‘AllPrep DNA/RNA Mini Kit’ (Qiagen) and cDNA was prepared using the ‘High Capacity cDNA Reverse Transcription Kit’ (Life Technologies). To find the most stable endogenous housekeeping genes, the GeNorm method was used [48]. Commercially available primers (TaqMan Gene Expression Assay; Life Technologies) for ELOVL1-7 (Hs00249277_m1, Hs00214936_m1, Hs00537016_m1, Hs00224122_m1, Hs01094704_m1, Hs00907564_m1, Hs00405150_m1), and the housekeeping genes ESD (Hs00382667_m1), HMBS (Hs00609297_m1), POLR2A (Hs00172187_m1), PSMB4 (Hs00160598_m1), TBP (Hs00427621_m1) and UBC (Hs00824723_m1) were used. qPCR was conducted on an OpenArray instrument (Applied Biosystems, Carlsbad, California, USA). qPCR was carried out on 30 SCC tissues and 9 pools (14-15 different samples) of matched normal tissues.

For cells and mouse tissue, RNA and cDNA were prepared using the ‘PureLink RNA Mini Kit’ (Life Technologies) and the ‘Superscript II Reverse Transcriptase’ (Life Technologies), respectively. qPCR was conducted on a FAST7500 AB system (Applied Biosystems) for humanELOVL1-7, mouseELOVL1-7 and 18S. The primers used are given in Supplementary Table S2.

### 2D-Imaging MS

MALDI (matrix-assisted laser desorption/ionization) imaging [49, 50] experiments were performed in positive ion mode as described previously [6] to screen for different PC lipid species. Briefly, all ion images were collected using a 15T MALDI FTICR mass spectrometer (Bruker Daltonics, Billerica, Massachusetts, USA) operated in positive ion mode with a spatial resolution of 100 μm and a mass resolution of 200,000 at *m/z* 734.6. Mass calibration was performed externally and all identifications were made using mass accuracy (<2 ppm).

### *In situ* hybridization (ISH)

Paraffin embedded tissue sections were stained for ELOVL6 (425441; Advanced Cell Diagnostics, Hayward, California, USA) using the RNAscope 2.0 Assay-RED detection kit (Advanced Cell Diagnostics). Slides were counterstained using hematoxylin and ammonia water. Images were taken on a Zeiss Axioplan 2 light microscope using a Plan-Neofluar 20x/0.5 objective. The acquisition software used was AxioVision 4.8.

### Statistical analysis

The results were analyzed by statistical tests available in GraphPad Prism (Version 6.0c). For every analysis, the specific statistical test used is indicated in the figure legend. * *p* < 0.05; ** *p* < 0.01; *** *p* < 0.001; **** *p* < 0.0001.

## SUPPLEMENTARY MATERIAL FIGURES AND TABLES



## Abbreviations

ACACA: acetyl-CoA carboxylase
AUC: area under the ROC-curve
ELOVL: elongation of very long fatty acids
ESI-MS/MS: electrospray ionization tandem mass spectrometry
IKKα: IκB Kinase α
MALDI: matrix-assisted laser desorption/ionization
MRM: multiple reaction monitoring
MS: mass spectrometry
NASH: nonalcoholic steatohepatitis
PC: phosphatidylcholine
PE: phosphatidylethanolamine
PI: phosphatidylinositol
PS: phosphatidylserine
SCC: squamous cell carcinoma
