# Supplementary material for: Phospholipid profiling identifies acyl chain elongation as a ubiquitous trait and potential target for the treatment of lung squamous cell carcinoma
Source: Oncotarget. 2016 Feb 3;7(11):12582–97. doi: 10.18632/oncotarget.7179 (PMC4914306; doi:10.18632/oncotarget.7179)
Supplement: Supplementary file 1 [file oncotarget-07-12582-s001.pdf]

Phospholipid profiling identifies acyl chain elongation as a ubiquitous trait and potential target for the treatment of lung squamous cell carcinoma

Supplementary Material

Table S1: Clinical characteristics of lung SCC patients

Variable	Statistic	Set 1	Set 2
Age	N	30	31
	Mean	63.9	64.1
	Std	9.76	8.66
	Median	62.5	64.0
	IQR	(57.0; 73.3)	(59.0; 70.0)
	Range	(43.0; 81.0)	(46.0; 80.0)
Sex			
Male	n/N (%)	25/30 (83.33%)	28/31 (90.32%)
Female	n/N (%)	5/30 (16.67%)	3/31 (9.68%)
Smoking status			
Smoker	n/N (%)	9/30 (30.00%)	17/31 (54.84%)
Non smoker	n/N (%)	0/30 (0.00%)	0/31 (0.00%)
StoppedS<6mo	n/N (%)	8/30 (26.67%)	2/31 (6.45%)
StoppedS>6mo	n/N (%)	13/30 (43.33%)	12/31 (38.71%)
Tumor stage			
I	n/N (%)	7/30 (23.33%)	8/31 (25.81%)
II	n/N (%)	9/30 (30.00%)	14/31 (45.16%)
III	n/N (%)	14/30 (46.67%)	9/31 (29.03%)

7th ed. of TNM classification was used. StoppedS<6mo = Stopped smoking less than 6 months; StoppedS>6mo = Stopped smoking more than 6 months

Table S2: Primers used for RT-qPCR

**hELOVL1**	Fw	CTGTGGCACAACCCTACCTT
	Rv	CTGGGAGATGTGCAGTGAGA
**hELOVL2**	Fw	TCTTTGGACCAACACTGAACA
	Rv	GTGATGGTGAGCACGAACTG
**hELOVL3**	Fw	CCTTGCAATCTTCAGTATCCT
	Rv	GATGATGAAGGCTGTGTCTC
**hELOVL4**	Fw	CCGAGAACCTTTTCAGATGC
	Rv	TCCACACTCTGGCAAATATAGC
**hELOVL5**	Fw	GTGCACATTCCCTCTTGGTT
	Rv	TTCAGGTGGTCTTTCCTTCG
**hELOVL6**	Fw	CAAAGCACCCGAACTAGGAG
	Rv	TGGTGATACCAGTGCAGGAA
**hELOVL7**	Fw	GCGCAAGAAAAATAGCCAAG
	Rv	GAATGTTCCCAAACCACCTG
**mELOVL1**	Fw	TTGGCTGAGTACCTACACCTG
	Rv	CTCGAACCATCCGAAGTGCTT
**mELOVL2**	Fw	AAGTCAATGCTTTCTTGGACAAC
	Rv	TACTTGTTACCCAGCCATATCG
**mELOVL3**	Fw	TTCTCACGCGGGTTAAAAATGG
	Rv	GAGCAACAGATAGACGACCAC
**mELOVL4**	Fw	GGGATCATACAACGCAGGATAC
	Rv	GTCCAAATACTCAACGCCTTTC
**mELOVL5**	Fw	GAACATTTCGATGCGTCACTCA
	Rv	GGAGGAACCATCCTTTGACTCTT
**mELOVL6**	Fw	GCAGAGAACACGTAGCGAC
	Rv	CGCAGAAAACAGGAAAGACTTC
**mELOVL7**	Fw	CATCGAGGACTGTGCGTTTTT
	Rv	CCAGGATGATGGTTTGTGGCA
**18S**	Fw	CGCCGCTAGAGGTGAAATTC
	Rv	TTGGCAAATGCTTTCGCTC

h = human primers; m = mouse primers

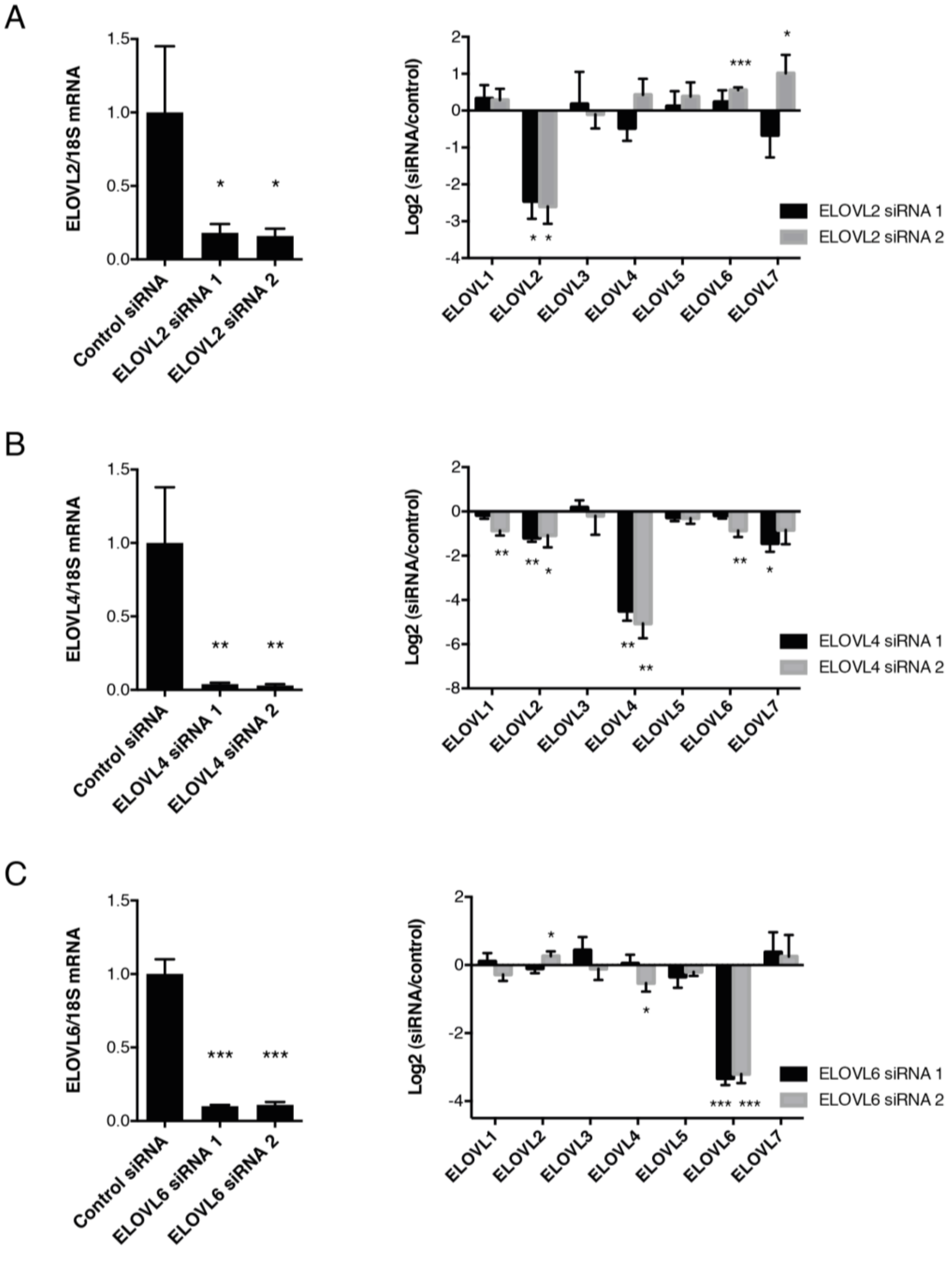

Figure S1: Effect of knockdown of ELOVL2, 4 and 6 on ELOVL expression in the SCC cell line 2427PT

2427PT cells were treated with two independent siRNAs specific for **(A)** ELOVL2, **(B)** ELOVL4 and **(C)** ELOVL6 for 48h. Cells treated with scrambled siRNA were used as a control. mRNA levels of ELOVL1-7 were analyzed by RT-qPCR. 18S was used for normalization. Data represent mean (n=3) ± standard deviation. * $p < 0.05$; ** $p < 0.01$; *** $p < 0.001$ (Student’s test).

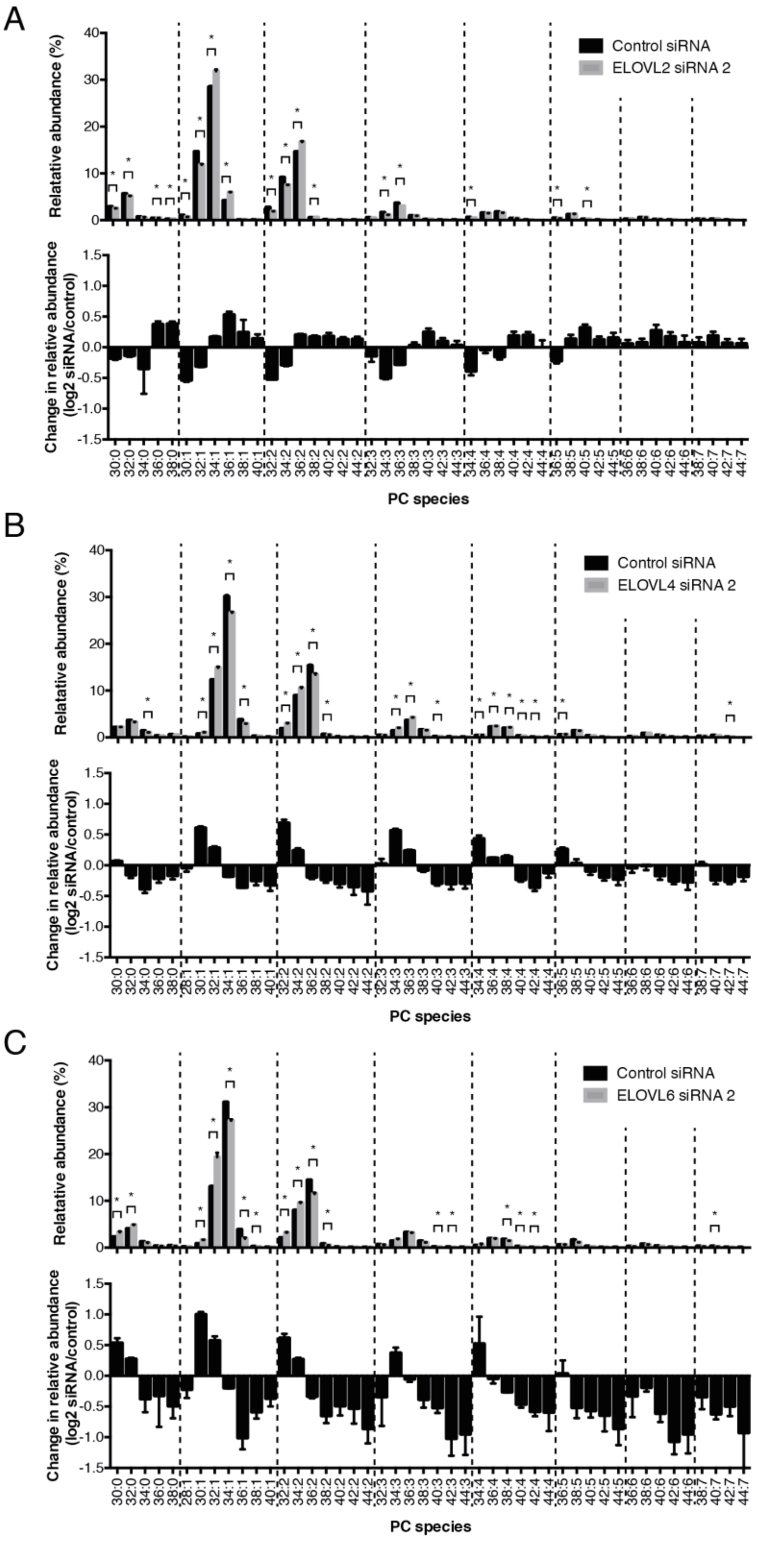

Figure S2: Effect of knockdown of ELOVL2, 4 and 6 by a second independent siRNA (siRNA 2) on PC profiles in 2427PT lung SCC cells

2427PT cells were transfected with a second siRNA specific for **(A)** ELOVL2, **(B)** ELOVL4 and **(C)** ELOVL6 for 72h. Control cells were transfected with scrambled siRNA. The relative abundance and changes in relative abundance (log2) of PC after modulation of the specific ELOVL are shown. Changes in lipids were analyzed and presented as in Figure 5. Data

represent mean (n=3) ± standard deviation. * Indicates statistical significance after multiple testing using the Holm-Sidak method with alpha=0.05.

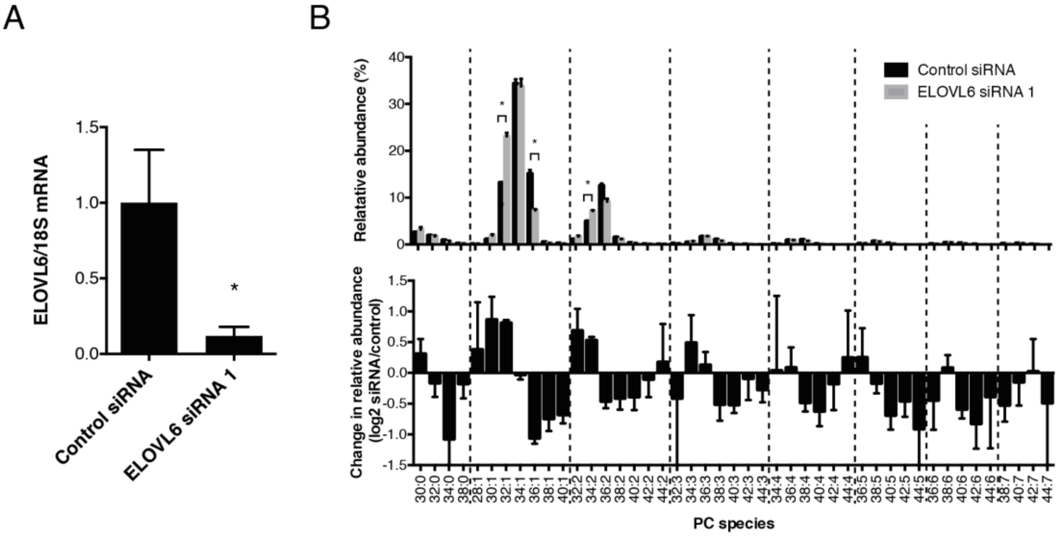

Figure S3: Effect of knockdown of ELOVL6 on acyl chain elongation in H2170 lung SCC cells

H2170 cells were treated with siRNA specific for ELOVL6 (siRNA 1) for **(A)** 48h and **(B)** 72h. Cells treated with scrambled siRNA were used as a control. **(A)** mRNA levels of ELOVL6 were analyzed by RT-qPCR. 18S was used for normalization. Data represent mean (n=2) ± standard deviation. * $p < 0.05$ (Student's test). **(B)** The relative abundance and changes in relative abundance (log2) of PC after modulation of ELOVL6 are shown. Changes in lipids were analyzed and presented as in Figure 5. Data represent mean (n=3) ± standard deviation. * Indicates statistical significance after multiple testing using the Holm-Sidak method with alpha=0.05.

Normal Tumor

		Normal				Tumor			
	**Species**	**%**	**Composition**	**% C16**	**% C18**	**%**	**Composition**	**% C16**	**% C18**
Patient 1	**PC34:3**	57.36	PC16:0/18:3	100.00	0.00	27.91	PC16:0/18:3	100.00	0.00
						33.33	PC18:3/16:0		
		42.64	PC16:1/18:2			38.76	PC16:1/18:2		
	**PC36:3**	31.71	PC16:0/20:3	36.90	63.10	36.20	PC16:0/20:3	42.41	57.59
		5.19	PC20:3/16:0			6.22	PC20:3/16:0		
		51.42	PC18:1/18:2			47.30	PC18:1/18:2		
		11.68	PC18:2/18:1			10.28	PC18:2/18:1		
	**PC38:3**	100.00	PC18:0/20:3	0.00	100.00	68.08	PC18:0/20:3	0.00	92.15
						8.36	PC18:1/20:2		
						15.71	PC20:2/18:1		
						7.85	PC20:1/18:2		
Patient 2	**PC34:3**			100.00	0.00	6.32	PC14:0/20:3	75.18	18.50
		50.58	PC16:0/18:3			17.38	PC16:0/18:3		
		12.19	PC18:3/16:0			11.52	PC18:3/16:0		
		29.00	PC16:1/18:2			30.48	PC16:1/18:2		
		8.23	PC18:2/16:1			15.80	PC18:2/16:1		
						8.62	PC18:1/16:2		
						9.87	PC16:2/18:1		
	**PC36:3**	37.63	PC16:0/20:3	40.50	59.50	36.45	PC16:0/20:3	45.04	54.96
		2.87	PC20:3/16:0			5.51	PC20:3/16:0		
						3.08	PC20:2/16:1		
		46.76	PC18:1/18:2			39.11	PC18:1/18:2		
		12.74	PC18:2/18:1			15.86	PC18:2/18:1		
	**PC38:3**	15.04	PC14:1/24:2	0.00	84.96			8.36	87.15
						4.43	PC16:0/22:3		
						3.93	PC22:3/16:0		
		66.55	PC18:0/20:3			54.43	PC18:0/20:3		
		18.41	PC20:3/18:0						
						6.89	PC18:1/20:2		
						25.83	PC20:2/18:1		
						4.49	PC20:1/18:2		
Patient 3	**PC34:3**	58.99	PC16:0/18:3	100.00	0.00	27.01	PC16:0/18:3	100.00	0.00
		26.88	PC16:1/18:2			29.42	PC16:1/18:2		
		14.13	PC18:2/16:1			43.57	PC18:2/16:1		
	**PC36:3**	37.34	PC16:0/20:3	42.03	57.97	53.65	PC16:0/20:3	60.25	39.75
		4.70	PC20:3/16:0			6.60	PC20:3/16:0		
		41.56	PC18:1/18:2			28.57	PC18:1/18:2		
		16.41	PC18:2/18:1			11.18	PC18:2/18:1		
	**PC38:3**	100.00	PC18:0/20:3	0.00	100.00	69.65	PC18:0/20:3	0.00	91.62
						8.30	PC18:1/20:2		
						13.67	PC20:2/18:1		
						8.38	PC20:1/18:2		

Figure S4: Fatty acyl composition of specific phospholipid species in 3 representative human SCCs and matched normal tissues

The molecular composition of PC species from tumor and matched normal tissues was characterized by MS3 fragmentation. % C16 represents the relative abundance of PC species containing C16:0 or C16:1 in one of the fatty acyl chains. Accordingly, % C18 represents the relative abundance of PC species containing C18:0 or C18:1 in one of the fatty acyl chains. Blue indicates a decrease in relative phospholipid abundance of that species in normal tissue

while red represents an increase in relative phospholipid abundance in tumor tissue as indicated by the scale bar.

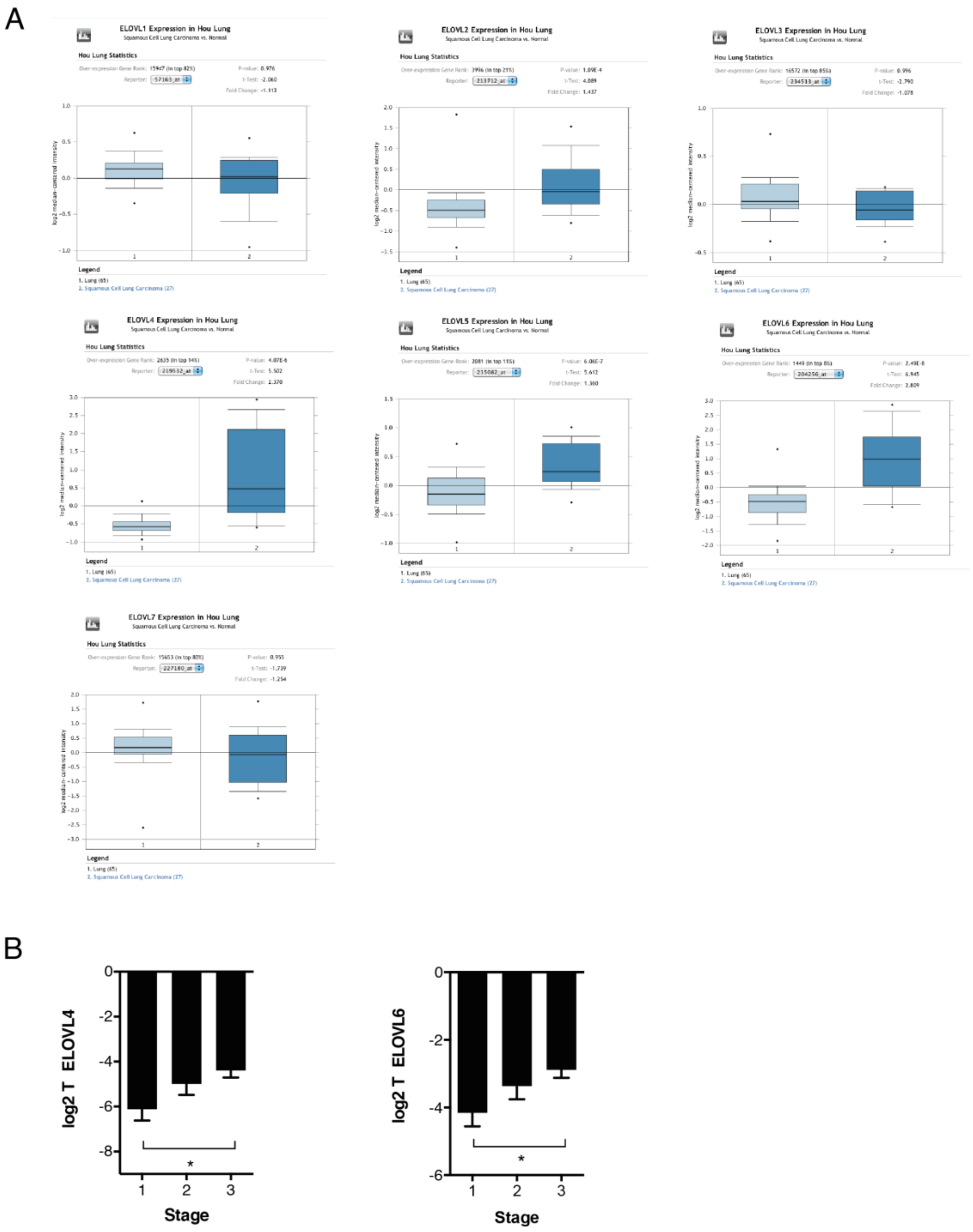

Figure S5: ELOVL6 mRNA expression in lung SCC in the Oncomine database (https://www.oncomine.org)

(A) Differences in ELOVL1-7 mRNA expression between SCC versus normal lung tissue were assessed in the Oncomine database [1] for dataset Hou *et al.* **(B)** Correlation of mRNA level of ELOVL4 and ELOVL6 with clinical stage in 61 human SCCs. * $p < 0.05$ (Kruskal-Wallis test/One-way ANOVA).

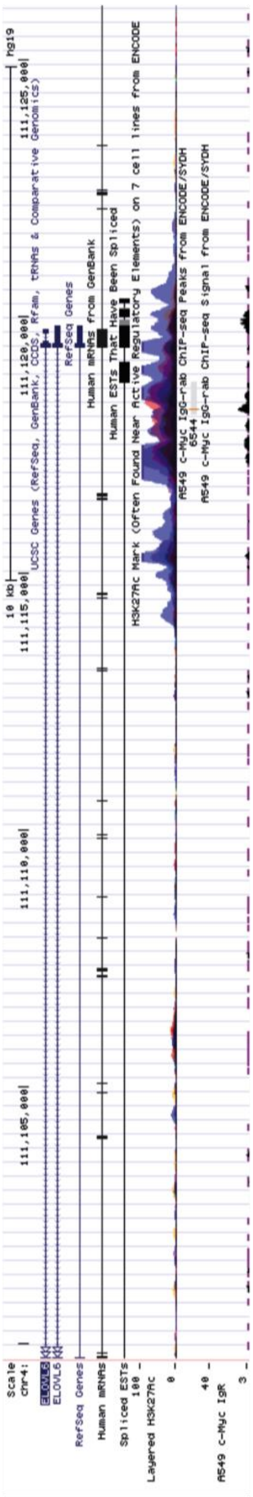

Figure S6: ChIP seq data for MYC in the lung cancer cell line A549 (http://genome.ucsc.edu)

Data were visualized using the UCSC genome browser on Human Feb. 2009 (GRCh37/hg19) Assembly [2]. (track name: A549 c-Myc IgG-rab ChIP-seq Peaks/Signal from ENCODE/SYDH)

Supplemental references

1. Rhodes DR, Yu J, Shanker K, Deshpande N, Varambally R, Ghosh D, Barrette T, Pandey A and Chinnaiyan AM. ONCOMINE: a cancer microarray database and integrated data-mining platform. Neoplasia. 2004; 6(1):1-6.

2. Kent WJ, Sugnet CW, Furey TS, Roskin KM, Pringle TH, Zahler AM and Haussler D. The human genome browser at UCSC. Genome Res. 2002; 12(6):996-1006.
